# Hydrogel Polyester Scaffolds via Direct-Ink-Writing of Ad Hoc Designed Photocurable Macromonomer

**DOI:** 10.3390/polym14040711

**Published:** 2022-02-12

**Authors:** Tiziana Fuoco, Mo Chen, Shubham Jain, Xi Vincent Wang, Lihui Wang, Anna Finne-Wistrand

**Affiliations:** 1 Department of Fibre and Polymer Technology, School of Engineering Sciences in Chemistry, Biotechnology and Health, KTH Royal Institute of Technology, Teknikringen, 56-58, SE 100-44 Stockholm, Sweden; shubhamj@kth.se (S.J.); annaf@kth.se (A.F.-W.); 2 Department of Production Engineering, School of Industrial Engineering and Management, KTH Royal Institute of Technology, Brinellvägen 68, SE 114-28 Stockholm, Sweden; wangxi@kth.se (X.V.W.); lihuiw@kth.se (L.W.)

**Keywords:** additive manufacturing, degradable polyesters, soft scaffolds, hydrophilic materials

## Abstract

Synthetic, degradable macromonomers have been developed to serve as ink for 3D printing technologies based on direct-ink-writing. The macromonomers are purposely designed to be cross-linkable under the radical mechanism, to impart hydrophilicity to the final material, and to have rheological properties matching the printer’s requirements. The suitable viscosity enables the ink to be printed at room temperature, in absence of organic solvents, and to be cross-linked to manufacture soft 3D scaffolds that show no indirect cytotoxicity and have a hydration capacity of up to 100% their mass and a compressive modulus in the range of 0.4–2 MPa.

## 1. Introduction

Aliphatic polyesters have a leading role among the polymeric biomaterials to manufacture 3D structures, defined as scaffolds, which serve as tridimensional support for tissue regeneration. The ability to degrade in the physiological environment and abiotic conditions is a great asset of polyesters. Additionally, polyester scaffolds provide mechanical support to the tissue while eroding over time, enabling tissue growth, and remodeling [1].

The traditional aliphatic polyesters, namely poly(lactide) (PLA), poly(ε-caprolactone) (PCL), poly(glycolide) (PGA), and relative copolymers, are thermoplastic materials that have been thermally processed to manufacture, for example, fibers for sutures and meshes [2,3]. Today, especially for PLA and PCL, they are used in melt-based additive manufacturing technologies, such as fused filament fabrication (FFF) to fabricate 3D printed scaffolds [4]. The physical properties and the chemical structure of the traditional aliphatic polyesters are suitable for additive manufacturing technology based on melt processing. However, the lack of thermal stability with melt processing represents a downside of the use of polyesters in melt-based additive manufacturing, which—as we have recently shown—can be overcome by the careful selection of processing methods and processing parameters [4,5,6], or by new material designs [7,8]. Furthermore, the elastic modulus of devices manufactured by using such polymers is usually in the range of GPa to Mpa, and these values are an order of magnitude higher than soft tissues, which have moduli ranging from kPa to a few MPa [9]. The hydrophobicity, the negligible water uptake of these materials, and the stiffness of the scaffolds, especially when PLA is used for printing, are also drawbacks for applications in soft tissue engineering.

Different from synthetic polyesters, natural polymers are printable at room temperature by a variety of additive manufacturing technologies and afford hydrogel scaffolds, which are hydrophilic and have a water content similar to native tissue. However, natural polymers do not measure up with synthetic polymers due to an inability in manipulating their properties and degradation profile and the variation that often occurs from batch to batch [9].

The need for expanding the library with synthetic and degradable polymers is recognized in the scientific community to engineer new printable materials for scaffold manufacturing while matching the requirements of both new applications, such as soft tissue engineering [9] and advanced manufacturing technologies [10]. Additive manufacturing technologies are burgeoning in the field of tissue engineering to fabricate 3D personalized scaffolds and implants with complex yet controlled geometry [11,12]. However, the fast and advanced development of additive manufacturing technologies corresponds to a lack of new, synthetic and printable polymeric materials to afford different types of scaffolds, such as hydrogel scaffolds.

Among 3D printing methods, technologies that involve the photo-polymerization of monomers and/or oligomers to produce 3D printed objects are particularly suitable for producing hydrogel scaffolds for soft tissue engineering [13,14].

The resin or ink for printing usually consists of unsaturated monomers or oligomers combined with an initiator [15]. The properties of the final 3D printed object are dictated by the structure of the printable monomers or oligomers.

Among the polyesters’ family, poly(ethylene glycol) [PEG]–poly(propylene fumarate) block copolymers have been recently developed as resin for continuous digital light processing (DLP) to manufacture soft scaffolds [16]. The combinations of PCL and chitosan have also been used as material for the DLP of scaffolds [17,18]. DLP techniques require, however, the use of a diluent or solvent, since the viscosity of the resin must be very low [19]. On the contrary, direct-ink-writing (DIW) methods require ink that is deposited layer-by-layer in a similar way to the molten filament in FFF, and the use of solvents and/or diluent can be avoided [20]. The ink must, however, have suitable rheological properties to allow printability: the material should be able to flow during the extrusion process while being self-supporting and retaining shape after printing [20,21]. The rheological properties of the material are indeed a crucial aspect to fulfilling the requirements of DIW methods. When acrylated [22] or functionalized polymers are used as ink, cross-linking reactions can occur while or after printing in the presence of a radical initiator triggered by UV radiation [23,24]. Thus, cross-linked polymeric networks are fabricated with a specific geometry, and gel scaffolds can be obtained [25]. DIW technologies are suitable for the fabrication of hydrogel-based devices [26,27,28] and biomedical applications [29].

**Figure 1:**
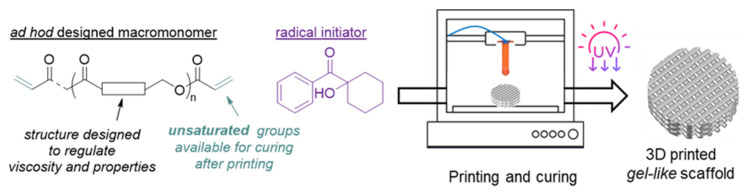
Overview of the approach.

Hence, the aim was to develop synthetic, degradable biomaterials that serve as a single-component ink for DIW technology and enable printing at room temperature—free from volatile organic solvents of polyester-based scaffolds that behave as hydrogels—and are suitable for soft tissue regeneration. To reach such an aim (i) a telechelic and cross-linkable macromonomer was designed and synthesized to have suitable rheological properties at room temperature to fulfill the requirements of the DIW technology; (ii) the DIW process was optimized; and (iii) 3D scaffolds were produced with properties suitable for soft tissue regeneration (Figure 1).

## 2. Materials and Methods

### 2.1. Materials

The oligomerization was performed in an MBraun glove box. Additionally, ε-caprolactone (CL) was purchased from Fisher Scientific and distilled in a vacuum over CaH_2_ before being stored in the glove box over 4 Å molecular sieves; *p*-dioxanone (DX) was purchased from Chemtronica and used as received. Diphenyl phosphate (DPP), polyethylene glycol 300 (PEG_300_), triethylamine (NEt_3_), acryloyl chloride, and 1-hydroxycyclohexyl phenyl ketone (Irgacure 184) were purchased from Sigma-Aldrich and used as received.

### 2.2. Polymer Synthesis and Chain-End Acrylation

The oligomerization reactions of CL and DX were performed in bulk under an inert atmosphere in a 50 mL round bottom flask equipped with a magnetic stir bar. The initiator, PEG_300_ (2 g, ca. 6.2 mmol of –OH groups) was added to the flask, followed by DX (6.02 g, 0.059 mol), CL (18.4 g, 0.161 mol), and DPP (350 mg, 1.07 mmol). The oligomerization reaction proceeded at room temperature for six hours. Afterward, the oligomer was purified by precipitation in 1 *w*/*w* % of a solution of pyridine in n-heptane; the precipitate was washed twice with n-heptane, dissolved in anhydrous CH_2_Cl_2,_ and transferred into a 250 mL Schlenk flask. The solvent was evaporated trap by trap and the oligomer was left to dry in a vacuum for 24 h. For the acrylation of the hydroxyl chain-ends, the oligomer was dissolved in 60 mL of anhydrous CH_2_Cl_2_ and the solution cooled down to 0 °C. NEt_3_ (1.70 mL, 0.012 mol) was added to the reaction mixture, followed by the drop-wise addition over 20 min of acryloyl chloride (1.40 mL, 0.017 mol). The reaction proceeded for 3 h while it slowly returned to room temperature. The mixture was then precipitated in n-heptane, and the precipitate was washed three times with cold methanol. The diacrylate oligomer was collected as a slightly yellow viscous product after drying in a vacuum oven for 76 h with a yield of 80%. *M*_n_ = 6800 g mol^−1^ (*Ð* = 1.2). Degree of acrylation = 70%.

^1^H NMR (400 MHz, CDCl_3_, rt) δ 6.37 (dd, *J* = 17.2, *J* = 17.2, 1.6, 1H, C*H*_2_=), 6.09 (dd, *J* = 17.2, *J* = 10.4, 1.6, 1H, =C*H*–), 5.80 (dd, *J* = 10.4, *J* = 1.6, 1H, C*H*_2_=), 4.34 (t, *J* = 4.2 Hz, 2H, -C(O)CH_2_OCH_2_C_2_O-,DX*-DX), 4.26 (t, *J* = 4.5 Hz, 2H, –C(O)CH_2_OCH_2_C*H*_2_O–, DX*-CL), 4.17-4.10 (overlapped signals: s, 2H, –C(O)C*H*_2_OCH_2_CH_2_O–; DX; t, *J* = 3.7 Hz, 2H, –C*H*_2_O–; CL*-DX), 4.04 (t, *J* = 6.7 Hz, 2H, –C*H*_2_O-; CL*-CL), 3.80–3.72 (m, 2H, –C(O)CH_2_OC*H*_2_CH_2_O–; DX), 3.60-3.70 (overlapped signals: 2H, –C*H*_2_OH; DX; t, *J* = 6.5 Hz, 2H, –C*H*_2_OH; CL and 4H –OC*H*_2_CH_2_O-), 2.35 (t, *J* = 7.5 Hz, 2H, –C(O)C*H*_2_–, CL*-DX), 2.29 (t, *J* = 7.4 Hz, 2H, –C(O)C*H*_2_–, CL*-CL), 1.69 –1.61 (m, 4H, –C(O)CH_2_C*H*_2_– and –CH_2_C*H*_2_O–; CL), 1.41–1.33 (m, 2H, -C*H*_2_-; CL).

^13^C NMR (100 MHz, CDCl_3_, rt) 173.8, 173.7, 173.65, 173.6, 173.5 and 173.45 (–*C*(O)O–; CL), 170.3, 170.25, 170.2 and 170.15 (–*C*(O)O–; DX), 130.7, 129.7 and 128.6 (*C*=), 70.7 and 70.65 (–C(O)CH_2_O*C*H_2_CH_2_O–; DX), 69.62, 69.4 and 69.3 (–C(O)*C*H_2_OCH_2_CH_2_O–; DX), 68.5, 68.4, 68.3 (–C(O)CH_2_OCH_2_*C*H_2_O–; DX), 64.8, 64.4, 64.25, 63.8, 63.4, 62.75 and 62.7 (–*C*H_2_O–; CL), 34.2, 34.15, 34.05 and 34.0 (–*C*H_2_C(O)O–; CL) 28.45, 28.4, 25.65, 25.6, 25.55, 25.5, 24.7, 24.6, 24.55 and 24.5 (–*C*H_2_–, CL).

### 2.3. Resin Preparation

The resin for 3D printing was prepared by softening the acrylated oligomer at 35 °C for 20 min and then mechanically mixed with the radical initiator Irgacure 184 (5 *w*/*w* % to the oligomer) to ensure a homogenous distribution of the two components. The mixture was cooled down to ca. 0 °C, degassed for 10 min, and loaded in the UV-shield printer cartridge (Cellink, Gothenburg, Sweden).

### 2.4. Printing

The scaffolds were printed by a Cellink Inkredible bioprinter (Gothenburg, Sweden) with a positioning precision (XY) of 10 µm; layer resolution (Z) of 100 µm; and a build volume of 130 mm × 80 mm × 50 mm. The printing parameters are listed in Table 1.

The printing parameters were selected based on our own preliminary experiments. The layer height was selected to be the same as the nozzle diameter to minimize the squeeze between layers while ensuring their bond. The printing speed was set to 2 mm/s to guarantee a homogeneous string diameter under the set air pressure. The fill density and fill pattern settings resulted in a grid shape as the scaffold.

Six cubic scaffolds (16 × 16 mm) and fifteen cylindrical scaffolds (diameter 16 mm) each composed of 6 layers, and each layer being 0.8 mm, were printed.

### 2.5. Cheracterization Methods

#### 2.5.1. NMR

NMR spectra were recorded in CDCl_3_ at room temperature on a Bruker Advance 400 spectrometer (^1^H: 400.13; ^13^C: 100.62 MHz, respectively). The resonances and coupling constants were reported in ppm (δ) and Hz (*J*), respectively. ^1^H NMR spectra were referenced to the residual solvent proton at δ 7.26 ppm; ^13^C NMR spectra were referenced to the ^13^C signal of CDCl_3_ at δ 77.16 ppm. Spectra were recorded using Bruker TopSpin v2.1 software (Billerica, MA, USA). Data processing was performed using MestReNova v9.0.0 software (Santiago de Compostela, Spain). The assignment of peaks was performed according to the literature.

#### 2.5.2. Size Exclusion Chromatography

Molar mass (*M*_n_ and *M*_w_) and dispersity (*Ð*) were measured by size exclusion chromatography (SEC). The measurements were performed at 35 °C on a GPCMAX system equipped with a Viscotek VE3580 RI detector and three columns, including one guard column (PLgel 5 μm Guard) and two linear columns (PLgel 5 μm Mixed-D) (Malvern Panalytical, Worcestershire, UK). The mobile phase was CHCl_3_ at a flow rate of 0.5 mL min^−1^. Narrow polystyrene standards with a molar mass ranging from 1200 to 940,000 g mol^−1^ were used as calibrants, and the flow rate fluctuations were corrected using toluene as the internal standard. The data were processed by OmniSEC v. 5.1 software.

#### 2.5.3. Rheology

The viscoelastic properties of the acrylated oligomer were characterized by using a DHR-2 (TA instruments, New Castle, DE, USA), stainless steel parallel plate (25 mm in diameter), and a Peltier plate using a gap size of 0.5 mm. Flow sweeps were performed at 22 °C from 0.1–100 s^−1^ and 10 data points were taken per decade. The collection time for each point varied from 1 s of equilibration time and 5 s of averaging time, needed to achieve a steady measurement, to 5 s of equilibration time and 30 s of averaging time.

Data were processed using TRIOS software (TA Instruments, New Castle, DE, USA) v.4.5.0.42498.

#### 2.5.4. Gel Content and Water Uptake

The swelling ratio and the gel content of the printed scaffolds were determined by immersing the specimens in 10 mL of CHCl_3_ for 24 h. Three samples were tested. The mass of the swollen scaffolds was measured to calculate the swelling ratio according to the equation:

$$swelling\;ratio\;\left(\%\right)=\frac{m_{s}-m_{0}}{m_{0}}\;100$$

where *m_s_* is the mass of the swollen specimen and *m*_0_ is the original mass.

Afterward, the scaffolds were dried in a vacuum for 48 h and the gel content was calculated as the percentage of residual mass versus the original mass:

$$gel\;content\;\left(\%\right)=\frac{m_{d}}{m_{0}}\;100$$

where *m_d_* is the mass of the dried specimen.

The water uptake of the scaffolds was analogously determined by immersing the samples in 10 mL of phosphate buffer saline (pH = 7.4) at 37 °C for 24 h. Three samples were tested. The mass of the swollen scaffolds was measured to calculate the water uptake according to the equation:

$$water\;uptake\;\left(\%\right)=\frac{m_{s}-m_{0}}{m_{0}}\;100$$

#### 2.5.5. Differential Scanning Calorimetry

Thermal analysis of the samples was performed by differential scanning calorimetry (DSC) in aluminium pans and a Mettler Toledo DSC 1 instrument calibrated using indium. Measurements were performed under a nitrogen flow with a heating rate of 10 K min^−1^ from −80 to 120 °C or to 200 °C.

#### 2.5.6. Mechanical Properties

Compression tests were performed on eight cylindrical scaffolds composed with a diameter of 16 mm, 50% infill density, composed of 6 layers, each of 0.8 mm height, and printed with a nozzle of 0.8 mm. Compression tests were performed at room temperature using an Instron 5944 mechanical testing machine, equipped with a 500 N load cell at a rate of 0.5 mm m^−1^ up to 60% of deformation. The compressive modulus was calculated from the initial linear region of the stress-strain curve.

#### 2.5.7. Cytocompatibility of the Scaffolds

The cytocompatibility of the scaffolds was assessed by using human dermal primary fibroblast cells (HDF; Thermo Scientific, Waltham, MA, USA). The cells were expanded in a T-75 culture flask in high glucose Dulbecco’s Modified Eagle Medium (DMEM; Gibco) with L-glutamine and pyruvate, and containing fetal bovine serum (10% *v*/*v*; Gibco) and antibiotics (1% *v*/*v*; Gibco). The culture flask was maintained at 37 °C in a humidified atmosphere (5% CO_2_). The cells were expanded till they reached 70–80% confluency and then, detached using trypsin-EDTA (0.025%; Gibco).

The indirect cytotoxicity tests were performed using the conditioned medium collected after the printed scaffolds had been soaked. Specifically, prior to collecting the conditioned medium, dialysis was performed to remove the left-over radical initiator. To do so, the scaffolds were packed in a dialysis bag and kept in deionized water for 3 days. Afterward, the scaffolds were sterilized using 70% ethanol for 15 min and then kept under ultraviolet for 1 h. Subsequently, they were washed three times with PBS and left in sterile water for 48 h to make sure that any residual ethanol was removed. Eventually, the sterile scaffolds were soaked individually in 20 mL of culture medium for 24 h at 37 °C in a CO_2_ incubator. The conditioned medium was then collected.

Simultaneously, 5 × 10^3^ cells were suspended in 400 µL of culture medium and seeded in a 48-well plate and left to attach for 24 h. The culture medium was then replaced with the conditioned medium. The cytocompatibility was assessed by exposing the cells to the conditioned medium for 1 and 3 days. Cells treated with the fresh culture medium were used as a control.

AlamarBlue dye (Thermo Scientific, Waltham, MA, USA) was used to evaluate cell viability following the protocol of the manufacturer. On each day, alamarBlue was added in 10% *v*/*v* each well, and fluorescence was recorded at 560/590 excitation/emission wavelengths. Cell morphology was visualized by fluorescence microscopy. Cells were fixed using a 3.7% formaldehyde solution for 20 min at room temperature and washed with PBS. Furthermore, cells were permeabilized using a Triton-100 for 10 min. The nuclei were stained by using DAPI (4′,6-diamidino-2-phenylindole; 14.3 mM; Thermo Scientific) for 15 min, and actin was stained by Alexa Fluor 546 (6.6 µM; Thermo Scientific) for 30 min at room temperature. Cells were then visualized using a fluorescence microscope (Nikon Ti-S). False-color and background corrections were made using ImageJ (NIH, Madison, WI, USA).

## 3. Results and Discussion

To develop and optimize the printing of soft and hydrophilic polymeric scaffolds that behave as hydrogels, macromonomers were purposely designed to be used as photocurable ink, having a pseudoplastic relationship between shear rate and viscosity. Such a relationship is crucial, as the polymeric resin needs to easily flow out from the printing nozzle while the print retains its integrity and stability on the printing bed. Among the polyester family, acrylated oligo(ε-caprolactone)s have been reported as macromonomers to prepare polymer networks [30] and/or scaffolds with different geometry by using various techniques [31,32]. However, such oligomers are semicrystalline at room temperature, and depending on the manufacturing technology, they require either a solvent or a temperature higher than room temperature for printing [31]. Therefore, they were not suitable for our purpose.

**Scheme 1:**
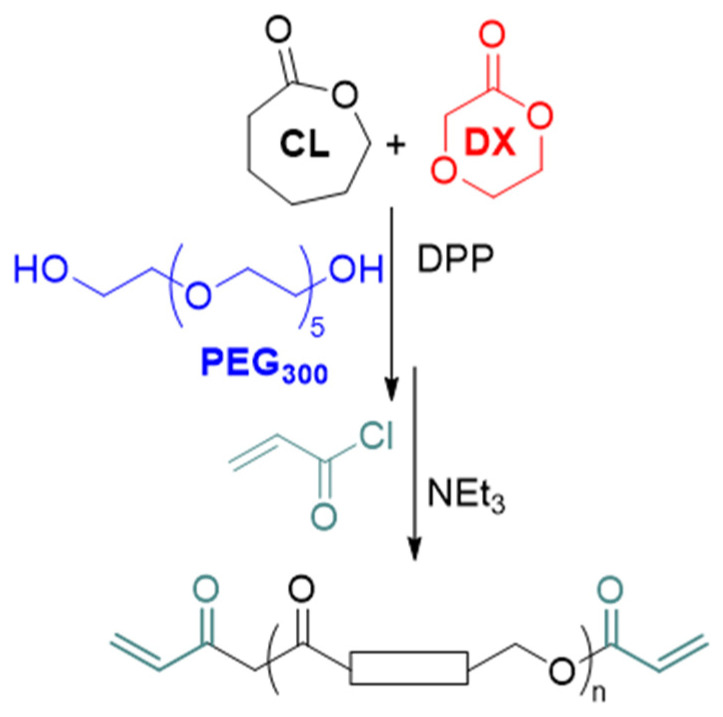
The rationale behind the ink design and synthesis.

Herein, the hypothesis was that acrylated co-oligomers of ε-caprolactone (CL) and *p*-dioxanone (DX), oligo(CL-co-DX), prepared by using a low molar mass polyethylene glycol (PEG_300_) as a difunctional initiator, could meet the aim if synthesized with a suitable molar mass, the composition of the two comonomers, and macromolecular architecture. Indeed, the combination of CL and DX enabled the preparation of high molar mass copolymers that are suitable for preparing scaffolds for soft tissue regeneration [7,8]. Here, by introducing a short PEG central block [33] and by increasing the amount of DX in the oligomers, materials were produced with higher hydrophilicity and behaved as hydrogels once cross-linked (Scheme 1).

The PEG_300_ central block would increase the hydrophilicity of the material, while the feed ratio of the two comonomers and the ratio of monomers to initiator would regulate the molar mass, and together with the PEG_300_ central block, dictate the viscoelastic, hydrophilicity, and thermal properties of the ink. The hydrolytic degradation of copolymers of CL and DX, even of a high molar mass, has been proved [7] and it has also been reported that PEG with a molar mass below 1 kg mol^−1^ is excreted within a few hours in humans [34]. Hence, the structure of the oligomers should also enable scaffold resorption.

The polymerization and chain-end modification were optimized based on our previous findings [35]. The photocurable macromonomers were prepared by co-oligomerization of CL and DX in the presence of PEG_300_ using diphenyl phosphate (DPP) as a catalyst, followed by acrylation of the chain-end (Full reaction scheme is reported in the Supplementary Information, Scheme S1). Specifically, the optimal properties for material printability (vide infra) were obtained when the ratio of CL to DX to PEG_300_ was 26 to 9.5 to 1. After 6 h of polymerization, the conversion of the monomers into oligomers was 86% for CL and 80% for the DX. The hydroxyl chain-ends were reacted with acryloyl chloride in the presence of NEt_3_ for 3 h to give the photocurable, telechelic macromonomers.

**Figure 2:**
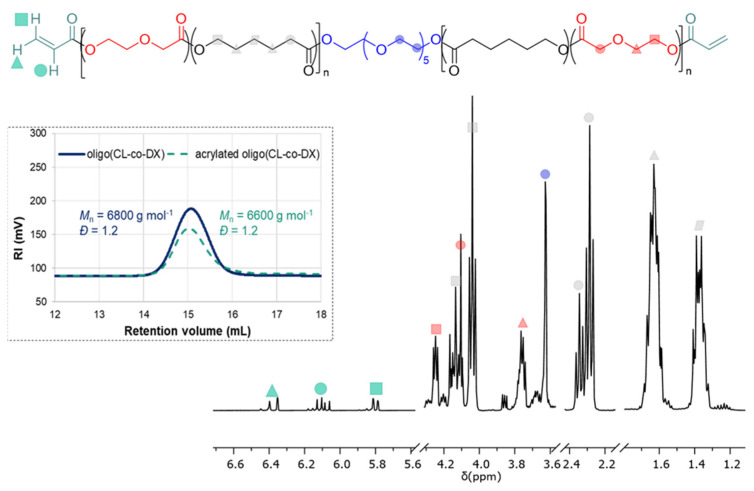
^1^H NMR spectra (CDCl_3_, 400 MHz) of the acrylated oligo(CL-co-DX). In the inset, SEC chromatograms of the oligo(CL-co-DX) and the acrylated oligo(CL-co-DX).

The ^1^H NMR spectra are reported in Figure 2.

Besides the signals of the CL and DX monomeric units in the range of 4.30–1.30 ppm and the PEG initiator at 3.65 ppm, the signals typical of the acryloyl moiety were detected at 6.37, 6.09, and 5.80 ppm, respectively. The ^1^H NMR confirmed that the resulting oligomers consisted of a central PEG block, which represents less than 10% in weight of the final co-oligomer, and two oligoester blocks with a random arrangement of CL and DX monomeric units. Furthermore, the signals at 4.10 and 4.04 ppm were both attributed to the ester methylene of the CL units (–C*H*_2_O–), analogously, the signals at 2.35 and 2.29 ppm were both assigned to the methylene next to the carbonyl moiety (–C(O)C*H*_2_–). Such patterns were indicative of the presence of both CL-CL homosequences and the CL*-DX heterosequence, which arise from the random distribution of the two monomeric units. The degree of acrylation of the hydroxyl chain-end was estimated to be 70 %. The calculation was performed by comparing the integral of the signal at 5.80 ppm of the acryloyl moiety and the integral of the signal at 2.40–2.20 ppm of the methylene group next to the carbonyl moiety of the CL units, assuming that each chain contained 21 CL units as a result of the stoichiometry of the reaction and monomer conversion (Scheme S1). The low degree of acrylation could be a consequence of the shorter reaction time, i.e., 3 h, and a lower temperature utilized than previous literature reports [31], which were however preferred to minimize side reactions, such as the hydrolysis of the ester bonds, and to prevent a decrease of the molar mass of the macromonomers and/or disruption of the telechelic structure. SEC analysis showed that the molar mass value of the acrylated oligomer was very similar to the pristine oligomer (Figure 2), thus, confirming that the reaction conditions used for the chain-end functionalization did not affect the oligomer structure and the unwanted degradation was minimized.

**Figure 3:**
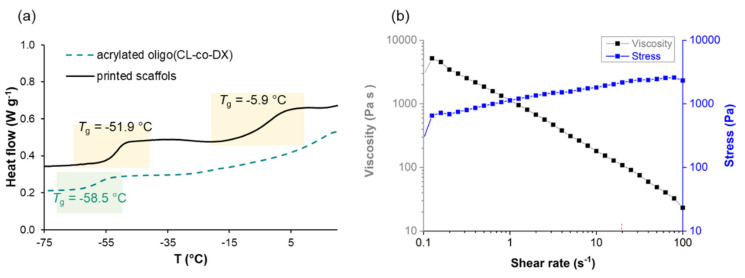
(**a**) DSC thermogram for the heating run, after cooling at −80 °C, of the acrylated oligo(CL-co-DX) and printed scaffolds. Endo peak up. (**b**) Representative flow curve of the acrylated oligo(CL-co-DX).

The acrylated oligo(CL-co-DX) was amorphous over the entire temperature range as observed by DSC analysis, with a *T*_g_ value of −58.5 °C (Figure 3a).

The rheological properties are those that mainly dictate materials’ printability. The decrease of viscosity with shear rate and the concomitant increase of the stress are typical of a pseudoplastic material. As previously reported [36] the shear rate γ ^·^ can be calculated with the equation:

γ ^·^ = 4Q/(πr^3^)

Q is the volumetric flow rate during extrusion and r is the radius of the nozzle. With a printing speed of 2 mm s^−1^ and a nozzle with an inner diameter of 0.8 mm, we estimated that the shear rate was 20 s^−1^ at the printing temperature of 22 °C, and hence the viscosity of the resin was ca. 108 Pa s according to Figure 3b. Thus, the as-synthesized oligomer is in the rubbery state at room temperature and the rheological tests showed that the material has, at this temperature, also suitable viscosity to fulfill the requirements of the printing technology we utilized (Figure 3b) [37].

**Figure 4:**
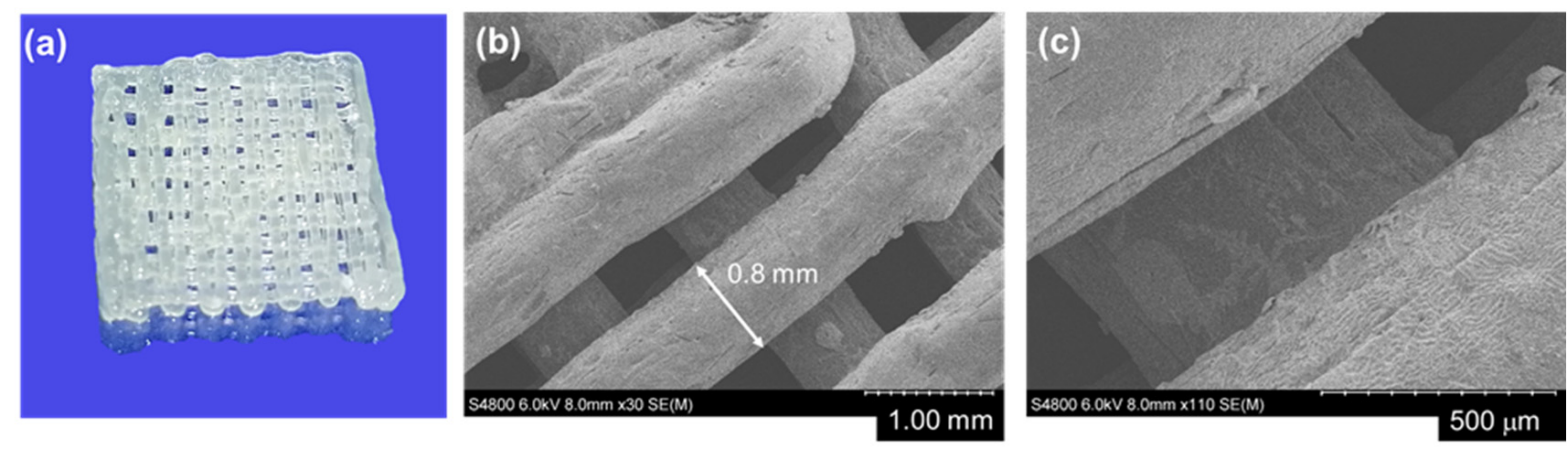
(**a**) Digital picture and (**b**,**c**) SEM images of the scaffold printed with a nozzle of 0.8 mm at a magnification of ×30 (**a**) and ×110 (**b**).

These proper viscoelastic properties enabled indeed the resin, composed of acrylated oligo(CL-co-DX) containing 5 *w*/*w* % of Irgacure 184 as radical initiator, to be extruded from the nozzle with a diameter of 0.8 mm at room temperature by applying a pressure of 200–220 kPa, while holding the desired structure (Figure 1 and Figure 4a).

The printing results were affected by various factors. In particular, it was observed that the following two factors had a noticeable impact on the structural integrity of printed scaffolds: (i) the presence of air bubbles in the ink and (ii) the number of layers of the scaffold. The air brought into the ink during the processing and loading of materials could cause intermittent extrusion, which generates voids in the strands of scaffolds. The problem was overcome by degassing the ink after it had been loaded into the printer cartridge. Due to the pressure of the above layers, it was noticed that the bottom layers of the scaffold tended to shrink along the gravity direction and even collapse as the printing process continued. Optimal results were achieved for continuous printing up to six layers for each scaffold, followed by curing of the structure. It was speculated that the printing of scaffolds with more than six layers would be achievable if the printing process is paused for curing of the lower printed layers, thereby ensuring their structural stability before printing the higher layers.

Thus, scaffolds with a height of up to six layers were printed without pausing the printing process for curing, and their photo cross-linking was performed by exposing the entire geometry of the scaffolds to UV light (365 nm) for 2 min, which showed a fast curing rate. The scaffolds had a thickness in the range of 3.4–3.5 mm and the UV intensity of the lamp, according to printer manufacturer specification, was ca. 3 mW cm^−2^.

Both cubic and cylindrical scaffolds were printed with precision over geometry. In Figure 4b,c, SEM images of the printed scaffold with a nozzle of 0.8 mm are shown. An exact correlation was measured between the nozzle size and the strand diameter for the top printed layer of the scaffolds, while some irregularities could be observed for the distance between the strands. This irregularity can be ascribed to both the printer, such as positioning errors of printer axes and the fluctuation of the printer’s air pressure, and to the inhomogeneity of ink. However, some slightly larger strands could be visually observed for the bottom layer.

The DSC thermogram of the cured scaffold showed the presence of two glass transitions, one occurring at a temperature of −5.9 °C and attributed to the cross-linked segments, which probably have reduced mobility compared to the uncross-linked oligomers, and another transition occurring at −51.9 °C, which is due to uncross-linked dangling chains (Figure 3a). The swelling ratio and gel content of the scaffolds were evaluated using chloroform as the solvent (Figure S1). The scaffolds had a high swelling capacity, up to (740 ± 100)% of their mass, while the gel content was about 70%. This value is in agreement with the degree of acrylation and thus, the presence of uncross-linked oligomers, as also indicated by thermal analysis. These results confirm that the cross-linking time of 2 min was enough to cure the acrylated oligomers since the gel content value agrees with the degree of acrylation of the chain-ends and indicate that the presence of uncross-linked oligomer was not due to the inefficiency of the curing process but to the lack of double bonds on a fraction of the chain-ends, which were, therefore, unable to be cross-linked.

The contact angle value of ca. 60°, measured on flat surfaces of cross-linked films of oligo(CL-co-DX), and the equilibrium water content of the scaffolds up to 100% their mass, confirmed the hydrophilic nature and the high wettability of the materials [38]. A high equilibrium water content (>70%) indicates that the material has a water content similar to native tissue, such as vasculature and skin. The elastic modulus of the scaffolds was evaluated in compression mode [39] at room temperature and in dry conditions, and it was estimated to be in the range of 0.4–2 MPa depending on the density of the scaffold (Figure S2). The density of the scaffolds, which was estimated by measuring the actual mass of each specimen and its volume, was simply varied by increasing the pressure during printing and therefore increasing the flow and the amount of material in the scaffolds’ volume. The manufactured scaffolds showed a high hydration capacity combined with good mechanical properties and can therefore mimic the properties of native soft tissues, such as cartilage and tendons [40,41]. The combination of both properties is difficult to be achieved with conventional hydrogels, which show moduli in the range of kPa when the water content is above 90% [42].

**Figure 5:**
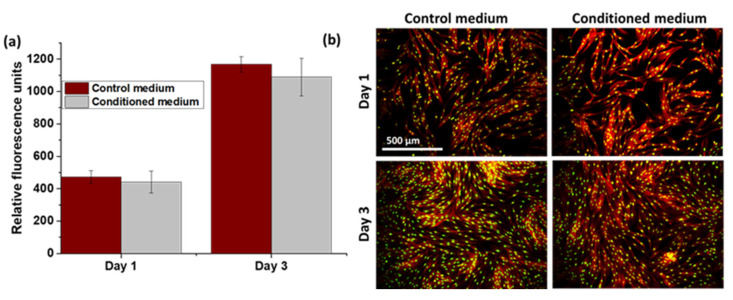
(**a**) AlamarBlue measurements of cell viability and proliferation at days 1 and 3 (**b**) Representative fluorescence micrographs at days 1 and 3. Green (nuclei) and Red (actin). ImageJ was used for better visualization of the cells.

Indirect cytotoxicity tests were performed over 3 days by using human dermal primary fibroblast cells (HDFs) and exposing them to a culture medium in which the scaffolds had been soaked for 24 h, named conditioned medium, as control cells treated with the fresh culture medium were used. Figure 5 shows representative results of the cell viability tests.

Before the cytotoxicity assessment, dialysis of the scaffolds was performed in deionized water to remove any residual of the radical initiator, which might induce reactive oxygen species and therefore affect cell viability [43]. The viability of the cells, determined using an alamarBlue assay, was comparable for cells cultured with conditioned media collected from scaffolds and cells cultured in fresh medium used as control (Figure 5a). This result indicates that no cytotoxicity was observed; indeed the number of cells significantly increased from day 1 to day 3 in both groups.

## 4. Conclusions

Three-dimensional printable aliphatic polyesters have been developed for direct-ink-writing technology. The ink was prepared by copolymerizing ε-caprolactone and *p*-dioxanone in presence of a short PEG as a bifunctional initiator. The acrylated oligomer combined with a radical initiator had suitable rheological properties to be printed at room temperature in a layer-by-layer fashion while retaining the desired structure. The cross-linking of the printed structure under UV light gave access to hydrogel scaffolds that do not affect cell viability and have a high hydration capacity and mechanical properties that can mimic the properties of soft native tissues.

These results give directions to overcome the dichotomy between the need to mimic the natural, soft tissue environment and scaffold degradability, which is not achievable with hydrogel-based scaffolds currently utilized. Further studies are ongoing to expand the printing technology scope and to utilize radical initiators that can be activated by visible light and are less harmful to the cells, to open the way to bioprinting.

## Figures and Tables

**Table 1:** Parameters for printing scaffolds.

Parameter Name	Value
Layer height	0.8 mm
Printing speed	2 mm s^−1^
Temperature	Room temperature (ca. 22 °C)
Nozzle diameter	0.8 mm
Air pressure	200–220 kPa
Fill density	50%
Fill pattern	Line

## Data Availability

The raw/processed data required to reproduce these findings cannot be shared at this time due to technical limitations. They are available on request.

## Supplementary Materials

The following supporting information can be downloaded at: https://www.mdpi.com/article/10.3390/polym14040711/s1, Scheme S1. Synthesis of the photocurable macromonomer by co-oligomerization of CL and DX using PEG as initiator and DPP as catalyst, followed by acrylation of the chain-end, Figure S1. (a) Water uptake, (b) contact angle and (c) gel content of the scaffolds. Pictures are representative of different samples and not in scale, and Figure S2. Plot of compressive modulus versus the density of the scaffolds.
