# Supplementary material for: Hydrogel Polyester Scaffolds via Direct-Ink-Writing of Ad Hoc Designed Photocurable Macromonomer
Source: Polymers (Basel). 2022 Feb 12;14(4):711. doi: 10.3390/polym14040711 (PMC8876641; doi:10.3390/polym14040711)
Supplement: Supplementary file 1 [file polymers-14-00711-s001.zip › polymers-1584799-supplementary.pdf]

SUPPLEMENTARY INFORMATION FOR:

Hydrogel Polyester Scaffolds via Direct-Ink-Writing of ad hoc Designed Photocurable Macromonomer

Tiziana Fuoco [1,*], Mo Chen [2,*], Shubham Jain [1], Xi Vincent Wang [2], Lihui Wang [2] and Anna Finne-Wistrand [1]

[1] Department of Fibre and Polymer Technology, School of Engineering Sciences in Chemistry, Biotechnology and Health, KTH Royal Institute of Technology, Teknikringen, 56-58, SE 100-44 Stockholm, Sweden; shubhamj@kth.se (S.J.); annaf@kth.se (A.F.-W.)

[2] Department of Production Engineering, School of Industrial Engineering and Management, KTH Royal Institute of Technology, Brinellvägen 68, S E 114-28 Stockholm, Sweden; wangxi@kth.se (X.V.W.); lihuiw@kth.se (L.W.)

* Correspondence: tiziana@kth.se (T.F.); mc3dp@outlook.com (M.C.)

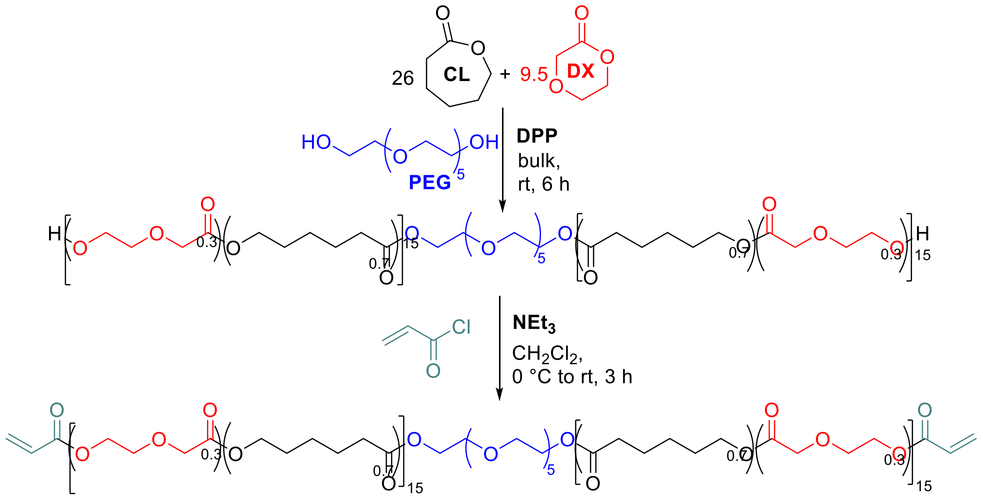

Scheme S1. Synthesis of the photocurable macromonomer by co-oligomerization of CL and DX using PEG as initiator and DPP as catalyst, followed by acrylation of the chain-end.

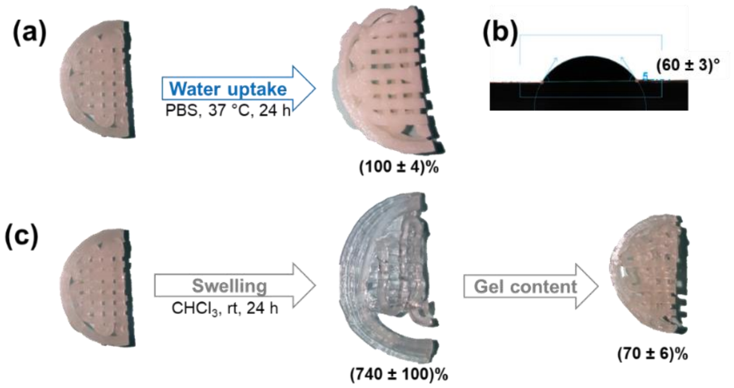

Figure S1. (a) Water uptake, (b) contact angle and (c) gel content of the scaffolds. Pictures are representative of different samples and not in scale.

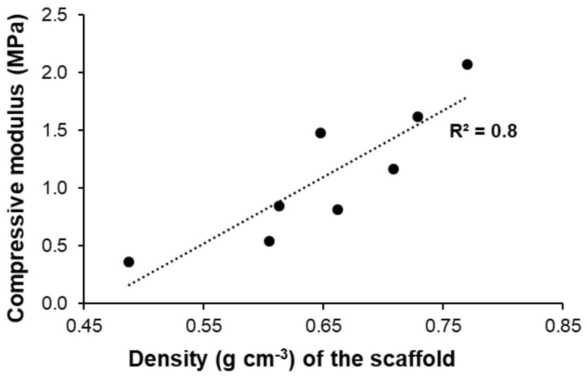

Figure S2. Plot of compressive modulus versus the density of the scaffolds.
